# Editorial for the Special Issue: “Therapeutic Drug Monitoring of Antimicrobials”

**DOI:** 10.3390/antibiotics11060815

**Published:** 2022-06-17

**Authors:** Matthias Gijsen, Karel Allegaert

**Affiliations:** 1 Clinical Pharmacology and Pharmacotherapy, Department of Pharmaceutical and Pharmacological Sciences, KU Leuven, 3000 Leuven, Belgium; karel.allegaert@uzleuven.be; 2 Pharmacy Department, UZ Leuven, 3000 Leuven, Belgium; 3 Department of Development and Regeneration, KU Leuven, 3000 Leuven, Belgium; 4 Department of Hospital Pharmacy, Erasmus MC University Medical Center, 3015 Rotterdam, The Netherlands

**Keywords:** therapeutic drug monitoring, precision dosing, antibiotic, antifungal, antiviral, dose optimization

A recent guideline [1] and position paper [2] defined dose optimization as a priority for antimicrobial research in special patient populations, such as critically ill patients. Therapeutic drug monitoring (TDM) is the most commonly recommended dose optimization strategy. TDM-based dose optimization strategies consist of evaluating the exposure—most commonly in the blood compartment—and adapting dosing based on predefined pharmacokinetic/pharmacodynamic targets. TDM results can be used as such, or they can be integrated in dosing nomograms or dosing software to optimize antimicrobial exposure, and ultimately, clinical outcome.

In the current Special Issue on “Therapeutic drug monitoring of antimicrobials”, several research articles and reviews have been published that support TDM-based antimicrobial dose optimization strategies in special patient populations. 

First, two research articles report on TDM-based dosing of vancomycin in two distinct populations. Vancomycin is an antimicrobial for which TDM is widely implemented and for which a clear exposure–outcome relationship has been defined.

In the first research article, Thijs and co-workers [3] show that a structured outpatient parenteral antimicrobial therapy (OPAT) program for vancomycin led to safe and effective ambulatory treatment of patients with continuous vancomycin infusion. A well-structured vancomycin OPAT program with bi-weekly follow-ups of plasma exposure led to therapeutic vancomycin exposure in the majority of the concentrations and ultimately led to clinical cure in all patients, with few adverse events. Additionally, good overall patient satisfaction was recorded. As such, Thijs and co-workers addressed the current paucity of data on the organization of an OPAT program for vancomycin and outcomes of such programs. In the future, patient burden could be further decreased by reducing the frequency of follow-ups, as data on the feasibility and safety of OPAT programs is accumulating.

In the second research article on vancomycin, Ueda and co-workers [4] retrospectively assessed the correlation between previously described vancomycin area-under-the-curve (AUC) cut-off values and clinical outcomes in patients with a methicillin-resistant *Staphylococcus aureus* (MRSA) infection. Vancomycin AUC was calculated using Bayesian estimation based on only one trough concentration. Ueda and co-workers were able to demonstrate higher early treatment response and higher early nephrotoxicity with trough-only estimated AUC values ≥400 µg × h/mL and ≥600 µg × h/mL, respectively (representing the previously recommended targets for efficacy and toxicity, respectively) [5]. However, in sub-analyses, these findings were only confirmed in patients receiving vancomycin twice daily (q12h) and in those with low-risk MRSA infections. This research illustrated that a trough-only approach might be used to perform Bayesian AUC-guided vancomycin dose optimization in perceived “low risk” populations. In contrast, this approach may not be precise or tailored enough for patients with moderate-to-severe MRSA infections and patients receiving vancomycin once daily. For these patients, a two-sample AUC estimation, or a one-sample AUC estimation based on concentrations taken earlier during the dosing interval, could be considered [6].

A third research article reported the development, validation and clinical application of a novel, simple and cost-effective method for the quantification of dalbavacin in human serum. Chiriac and co-workers [7] developed a high-performance liquid chromatography–ultraviolet spectrometry (HPLC–UV) method with acceptable bias and precision, and a clinically relevant quantification range. Dalbavacin is a new and promising alternative for the outpatient treatment of Gram-positives due its long half-life. In contrast to the only previously developed method for dalbavancin quantification, which relies on mass spectrometry, their method can be easily implemented in smaller laboratories. As such, the developed HPLC–UV method, with a short total processing time of approx. 20 min, can be used for routine TDM of dalbavancin. Interestingly, Chiriac and co-workers validated their method in three clinical cases, illustrating its potential clinical application for TDM-based dose optimization of dalbavancin in special patient populations. This paper hereby serves as an illustration of how compound-specific method development should be reported to provide sufficient confidence for researchers and care providers to apply this methodology in their clinical setting. 

The current Special Issue also consists of two reviews highlighting the potential of TDM-based dose optimization for antimicrobials.

Armengol and co-workers [8] assessed the current evidence on the pharmacology and pharmacokinetics of clindamycin, while identifying knowledge gaps to inform potential dose optimization strategies. In their review, important knowledge gaps regarding the pharmacokinetics and pharmacodynamics of clindamycin and its active metabolites were uncovered. They identified special patient populations with altered clindamycin pharmacokinetics, such as pediatric patients and pregnant and lactating women. Additionally, they uncovered several potential drug–drug interactions via the cytochrome P450 system that need to be addressed. Overall, there is a paucity of data on the pharmacokinetics, efficacy and safety of clindamycin and the contribution of its metabolites. These knowledge gaps need to be addressed before clindamycin dose optimization strategies can be investigated in the above-mentioned special patient populations.

Finally, in their review, Baracaldo-Santamaría and co-workers [9] highlighted the importance of TDM-based antifungal dose optimization. They provided an overview of the current evidence for TDM of the most commonly used antifungal drugs in critically ill patients. Moreover, for each class, a detailed summary of the evidence was provided regarding the pharmacokinetic/pharmacodynamic index and factors influencing the exposure. For the azoles, TDM is mainly considered for itraconazole, posaconazole and voriconazole. In addition to target attainment, the need for azole TDM is mainly driven by risk of toxicity and high probability of drug–drug interactions. Nevertheless, there have been reports that TDM of azoles, such as fluconazole, may also be beneficial to avoid underexposure [10]. Echinocandins, although generally considered safe, should also be considered candidates for TDM, as suboptimal exposure has been documented in critically ill patients. In contrast, for amphotericin B, routine TDM is currently not recommended, as its pharmacokinetic/pharmacodynamic relation is still not well understood and is yet to be elucidated. For flucytosine, routine TDM is recommended because of the highly variable exposure and severe toxicity associated with overexposure.

To conclude, the research articles and reviews provided in this Special Issue highlight the need for TDM-based dose optimization of several antimicrobials in special patient populations. For both antibiotics and antifungals, recommendations and areas for further research have been identified. However, the literature on therapeutic targets for antiviral therapy remains scarce. As a result, the potential benefit of antiviral TDM remains inconclusive [2]. Additionally, for many antimicrobials, the link between exposure and clinical outcome remains to be confirmed. These are areas, which necessitate future work, and ultimately, large studies to confirm the benefit from optimized dosing strategies. The topic of TDM therefore remains of utmost relevance to the overarching topic of this journal. Consequently, we intend to keep this Special Issue open for submissions as a tool to communicate and interact between clinical researchers.
